# Expressions of Fas ligand and other apoptosis-related genes and their prognostic significance in epithelial ovarian neoplasms

**DOI:** 10.1054/bjoc.1999.1073

**Published:** 2000-03-21

**Authors:** S Munakata*, T Enomoto, M Tsujimoto, Y Otsuki, H Miwa, H Kanno, K Aozasa

**Affiliations:** Departments of Pathology and Obstetrics and Gynecology, Osaka University Medical School, 2-2 Yamadaoka, Suita, Osaka, 565-0871, Japan; Pathology and Obstetrics and Gynecology, Osaka Police Hospital, 10-31 Kitayamacho, Tennoji-ku, Osaka, 543-0035, Japan

**Keywords:** Fas ligand, CD95 ligand, bcl-2, epithelial ovarian neoplasms, immunohistochemistry, prognosis

## Abstract

**Summary**Expression of apoptosis-related proteins, bcl-2, Bax, Fas and Fas ligand (L), in ovarian epithelial neoplasms together with its clinical relevance was examined by immunohistochemistry. They included 36 cases with adenoma, 33 with low potential malignancy (LPM) and 63 with carcinomas. bcl-2 expression was observed in 14 of 36 cases (39%) with adenoma, five of 33 (15%) with LPM (*P*< 0.05) and 12 of 63 (19%) with carcinoma (*P*< 0.05). Cases with bcl-2 expression showed more favourable prognosis than those without, but the difference was not statistically significant. There was no difference in frequency of Bax and Fas expression between each histologic category. Fas L expression was observed in one of 36 cases (3%) with adenoma, but in 12 of 33 (36%) with LPM (*P*< 0.001) and 42 of 63 (67%) with carcinoma (*P*< 0.0001). In carcinomas, cases expressing Fas L showed a less favourable prognosis than those without (*P*= 0.02). Density of CD8+ lymphocytes, possibly cytotoxic T-cells, was higher in serous carcinoma with negative Fas L expression than those with positive Fas L expression. These findings suggest that Fas L expressing carcinomas induce apoptosis in infiltrating CTL with Fas expression, and escape from immune surveillance. © 2000 Cancer Research Campaign


British Journal of Cancer (2000) **82**(8), 1446–1452
© 2000 Cancer Research Campaign
DOI: 10.1054/ bjoc.1999.1073, available online at http://www.idealibrary.com on IDEAL®

### Expressions of Fas ligand and other apoptosis-related genes and their prognostic significance in epithelial ovarian neoplasms

**S Munakata[1,*] T Enomoto[2], M Tsujimoto[3], Y Otsuki[4], H Miwa[1], H Kanno[1] and K Aozasa[1]**

Departments of [1]Pathology and [2]Obstetrics and Gynecology, Osaka University Medical School, 2-2 Yamadaoka, Suita, Osaka 565-0871, Japan; Departments of [3]Pathology and [4]Obstetrics and Gynecology, Osaka Police Hospital, 10-31 Kitayamacho, Tennoji-ku, Osaka 543-0035, Japan

**Summary** Expression of apoptosis-related proteins, bcl-2, Bax, Fas and Fas ligand (L), in ovarian epithelial neoplasms together with its clinical relevance was examined by immunohistochemistry. They included 36 cases with adenoma, 33 with low potential malignancy (LPM) and 63 with carcinomas. bcl-2 expression was observed in 14 of 36 cases (39%) with adenoma, five of 33 (15%) with LPM ($P < 0.05$) and 12 of 63 (19%) with carcinoma ($P < 0.05$). Cases with bcl-2 expression showed more favourable prognosis than those without, but the difference was not statistically significant. There was no difference in frequency of Bax and Fas expression between each histologic category. Fas L expression was observed in one of 36 cases (3%) with adenoma, but in 12 of 33 (36%) with LPM ($P < 0.001$) and 42 of 63 (67%) with carcinoma ($P < 0.0001$). In carcinomas, cases expressing Fas L showed a less favourable prognosis than those without ($P = 0.02$). Density of CD8+ lymphocytes, possibly cytotoxic T-cells, was higher in serous carcinoma with negative Fas L expression than those with positive Fas L expression. These findings suggest that Fas L expressing carcinomas induce apoptosis in infiltrating CTL with Fas expression, and escape from immune surveillance. © 2000 Cancer Research Campaign

**Keywords**: Fas ligand; CD95 ligand; bcl-2; epithelial ovarian neoplasms; immunohistochemistry; prognosis

Ovarian cancer is less prevalent in Japan than in Western countries, although the number of patients with ovarian cancer has been increasing in Japan (Barber, 1993). In both areas, surface epithelial-stromal tumours are the commonest, comprising about 60% of all ovarian neoplasms or over 80% of primary ovarian malignancies (Katsube et al, 1982; Koonings et al, 1989). Epithelial ovarian cancers have the poorest prognosis among gynaecological malignancies (Brinton and Hoover, 1992). To establish appropriate therapeutic modalities in these tumours, information for factors affecting prognosis is essential. Factors reported to influence the prognosis include performance status, age of patients, cell type (mucinous and clear cell), stage of disease, clinically measurable disease, tumour volume, presence or absence of ascites, tumour ploidy, expression of platelet-derived growth factor, HER-2/neu, or Ki-67 counts (Rodenburg et al, 1987; Slamon et al, 1989; Omura et al, 1991; Henriksen et al, 1993; Garzetti et al, 1995).

Bcl-2 is an oncoprotein known to inhibit programmed cell death (McDonnell et al, 1989; Hockenbery et al, 1990), and expression of this protein was correlated with favourable prognosis in lung, breast and ovarian cancers (Pezella et al, 1993; Joensuu et al, 1994; Henriksen et al, 1995). Bax, one of the bcl-2 superfamily proteins, induces apoptosis via heterodimerization with bcl-2, and absence or diminished expression of Bax gene in neuroblastoma (Hoehner et al, 1997) and neuroendocrine tumours of lung resulted in longer survival (Brambilla et al, 1996).

Fas (CD95), a member of the tumour necrosis factor (TNF) receptor family, is weakly expressed in many tissues, but strongly expressed in the human liver, heart, lung, kidney, ovary and activated lymphocytes (Nagata and Golstein, 1995). On the other hand, Fas ligand (Fas L/CD95 L) expression is observed in activated T- and NK-cells (Tanaka et al, 1996). Various cancer cell lines also express Fas L and induce Fas-mediated apoptosis in vitro in B-cell and T-cell lymphoma cell lines. Because Fas L expressions in vivo are reported in human carcinomas including melanoma, hepatocellular carcinoma, lung carcinoma, colon carcinoma, non-Hodgkin's lymphomas, oesophageal carcinoma and pancreatic adenocarcinoma (Hahne et al, 1996; Strand et al, 1996; Niehans et al, 1997; Shiraki et al, 1997; Bennett et al, 1998; Müllauer et al, 1998; Ungefroren et al, 1998), a similar mechanism might work in vivo, resulting in evasion of human tumours from immune surveillance.

In the current study, protein expression of Fas and Fas L together with bcl-2 and bax genes are examined in epithelial ovarian neoplasms including adenoma, tumour with low potential malignancy and carcinoma. Prognostic significance of the findings was evaluated.

### PATIENTS AND METHODS

A total of 132 epithelial ovarian neoplasms, including 36 adenomas (25 serous, ten mucinous and one Müllerian), 33 tumours of low potential malignancy (LPM) (14 serous, 17 mucinous and two Müllerian) and 63 carcinomas (33 serous, five mucinous, 13 endometrioid and 12 clear cell), were retrieved from the surgical pathology files of Osaka University Hospital (OUH) and Osaka Police Hospital (OPH). These patients were admitted to the hospitals during the period from June 1991 to November 1997 (OUH), and March 1980 to December 1997 (OPH). Brief clinical data were available in all patients together with follow-up data in 38 with carcinoma (OPH). In 35 carcinoma patients with adequate

*Received 20 April 1999*
*Revised 16 November 1999*
*Accepted 24 November 1999*

*Correspondence to:* S Munakata, Department of Pathology, Kansai Rosai Hospital, 3-1-69 Inabasou Amagasaki, Hyogo 660–8511, Japan

**Table 1** Modalities of surgery and chemotherapy in patients with ovarian carcinoma (*n* = 35)

| Fas L expression | Stage | Number of patients | Methods of surgery (Number of patients) | Chemotherapy | |
|---|---|---|---|---|---|
| | | | | Pre-operative | Post-operative |
| Fas L-positive (*n* = 22) | 1 | 7 | STH + SO (6)<br>SRH + LN (1) | 0 | PAC (7) |
| | 2 | 2 | STH + SO (2) | 0 | PAC (1)<br>FAM (1) |
| | 3 | 10 | STH + SO (5)<br>SRH (1)<br>RH (1)<br>Total reduction (1)<br>SO (1)<br>PL (1) | 2 | PAC (9)<br>Etoposide (1) |
| | 4 | 3 | STH + SO (1)<br>SCH + SO (2) | 2 | PAC (3) |
| Fas L-negative (*n* = 13) | 1 | 7 | STH + SO (7) | 0 | PAC (5)<br>MMC (1)<br>None (1) |
| | 2 | 2 | STH + SO (2) | 0 | PAC (2) |
| | 3 | 3 | STH + SO (3) | 1 | PAC (2)<br>CP (1) |
| | 4 | 1 | PL (1) | 0 | PAC (1) |

STH: simple total hysterectomy, SO: salpingo-oophorectomy, SRH: semiradical hysterectomy, RH: radical hysterectomy, SCH: supracervical hysterectomy, PL: probe laparotomy, PAC: cisplatin, doxorubicin and cyclophosphamide, CP: cisplatin and cyclophosphamide, FAM: 5-Fluorouracil, doxorubicin and mitomycin C, MMC: mitomycin C.

**Table 2** Clinicopathological findings in carcinoma patients

| Description | All (*n* = 35) | Fas L-positive (*n* = 22) | Fas L-negative (*n* = 13) | Statistics |
|---|---|---|---|---|
| Age | 53.1 ± 11.2 | 54.4 ± 12.0 | 50.9 ± 9.6 | NS |
| Follow-up period (months) | | | | |
| Range | 5–110 | 6–78 | 5–110 | |
| Median | 32 | 31.5 | 32.0 | |
| Mean | 41.3 | 39.0 | 45.3 | |
| Histology (No. of patients) | | | | |
| Serous | 17 | 13 | 4 | |
| Mucinous | 2 | 0 | 2 | |
| Endometrioid | 8 | 3 | 5 | |
| Clear cell | 8 | 6 | 2 | |
| Clinical stage (No. of patients) | | | | |
| I | 14 | 7 | 7 | |
| II | 4 | 2 | 2 | |
| III | 13 | 10 | 3 | |
| IV | 4 | 3 | 1 | NS |

NS: not significant.

clinical information, all patients received surgery; total hysterectomy with salpingo-oophorectomy (SO) in 26 cases, semiradical hysterectomy in two, supracervical hysterectomy with SO in two, probe laparotomy in two, and SO, radical hysterectomy and volume reduction surgery in one each (Table 1). Of these 35 patients, all but one received chemotherapy; PAC (cisplatin, doxorubicin and cyclophosphamide) in 30, CP, FAM (5-fluorouracil, doxorubicin and mitomycin C), etoposide and mitomycin C in one each. Five patients received chemotherapy prior to operation. There are no significant differences in modalities of surgery and chemotherapy between Fas L-positive and negative groups. Clinical parameters including age and clinical stage are shown in Table 2, showing no significant differences between Fas L-positive and -negative groups.

Histologic specimens were fixed in 10% formalin and routinely processed for paraffin embedding. Histologic sections, cut at 3 μm, were stained with haematoxylin and eosin and immunoperoxidase procedures. Histological subclassification and grading were performed by two pathologists (SM, MT).

### Immunohistochemistry

Sections were deparaffinized in three changes of d-limonene (Hemo De) (Fisher Chemical, PA, USA). After microwave oven heating (100°C, 15 min) with H2800 Microwave Processor (Energy Beam Sciences Inc., MA, USA) in citrate buffer (pH 6.0), sections were processed with use of LSAB kit (Dako, Kyoto, Japan). Primary monoclonal antibodies used were anti-bcl-2

*© 2000 Cancer Research Campaign* 

**Table 3** Summary of immunohistochmical studies

| | Number of positive cases (%) | | | |
|---|---|---|---|---|
| | Bcl-2 | Bax | Fas | Fas L |
| Adenoma | 14/36 (39) | 27/36 (75) | 28/36 (78) | 1/36 (3) |
| LPM | 5/33 (15) | 29/33 (88) | 27/33 (82) | 12/33 (36) |
| Carcinoma | 12/63 (19) | 47/63 (75) | 43/63 (68) | 42/63 (67) |

Bcl-2: Adenoma vs LPM, a; Adenoma vs Carcinoma, a. Fas L: Adenoma vs LPM, c; LPM vs Carcinoma, b; Adenoma vs Carcinoma, d.

[a]$P < 0.05$, [b]$P < 0.01$, [c]$P < 0.001$, [d]$P < 0.0001$.

**Table 4** Results of immunohistochemical study according to histological subtype

| | | Number of positive cases (%) | | | |
|---|---|---|---|---|---|
| Classification | Type | Bcl-2 | Bax | Fas | Fas L |
| Adenoma | Serous | 14/25 (56) | 20/25 (80) | 19/25 (76) | 1/25 (4) |
| | Mucinous | 0/10 (0) | 7/10 (70) | 8/10 (80) | 0/10 (0) |
| | Müllerian | 0/1 (0) | 0/1 (0) | 1/1 (100) | 0/1 (0) |
| LPM | Serous | 4/14 (29) | 14/14 (100) | 11/14 (79) | 9/14 (64) |
| | Mucinous | 0/17 (0) | 14/17 (82) | 14/17 (82) | 3/17 (18) |
| | Müllerian | 1/2 (50) | 1/2 (50) | 2/2 (100) | 0/2 (0) |
| Carcinoma | Serous | 3/33 (9) | 24/33 (73) | 23/33 (70) | 25/33 (76) |
| | Mucinous | 0/5 (0) | 2/5 (40) | 3/5 (60) | 1/5 (20) |
| | Endometrioid | 3/13 (23) | 10/13 (77) | 10/13 (77) | 7/13 (54) |
| | Clear cell | 6/12 (50) | 11/12 (92) | 7/12 (58) | 9/12 (75) |

Bcl-2: Adenoma serous vs mucinous, a; LPM serous vs mucinous, b; Carcinoma serous vs clear cell, a. Fas L: LPM serous vs mucinous, b.

[a]$P < 0.01$, [b]$P < 0.05$.

(Dakopatts, Glostrup, Denmark, diluted at 1:100), anti-Fas (UB-2; MBL, Japan, diluted at 1:25), and anti-Fas L (clone 33; Transduction Laboratories, KY, USA, diluted at 1:50), together with polyclonal anti-Bax (Dakopatts, diluted at 1:50) antibody. 2′,5′-Diaminobenzidine (DAB) was used as a chromogen. Normal tonsils were obtained as positive control for bcl-2 and Bax. Tonsils were fixed in 10% buffered formalin and processed routinely. Jurkat cells (gift from Dr M Nose, Matsuyama, Japan), used as a positive control for Fas, were fixed in 10% buffered formalin after centrifugation and routinely processed for paraffin embedding. As a Fas L-positive control, Phytohaemagglutinin (PHA) activated Jurkat cells were used. A total of $1 \times 10^7$ Jurkat cells were cultivated and fixed in 10% buffered formalin after incubation in 2% PHA (Life Technologies, Tokyo, Japan) at 37°C for 5 h. For negative control, $IgG_1$ isotype (Dakopatts, diluted at 1:25) was used as a primary antibody on each section. Some of the specimens were treated with rabbit polyclonal anti-Fas L (C-20, Santa Cruz Biotechnology, Santa Cruz, CA, USA, diluted 1:100) raised to residues 260–279 of human Fas L, to confirm the specificity of monoclonal anti-Fas L (clone 33). Both antibodies showed the comparable results (data not shown).

Numbers of positively stained cells with each antibody (P) were scored as 0 (0%), 1 (< 10%), 2 (10–50%), or 3 (> 50%). Staining intensity (I) was graded semiquantitatively as 0 (none), 1 (weak), 2 (moderate), or 3 (intense). Then values of $P \times I$ (PI) with $\geq 2$ were regarded as positive.

Degree of lymphocytic infiltration in the tumour tissue was graded as 0 (none), 1 (scarce), 2 (moderate) and 3 (massive). Mean score of lymphocyte infiltration index (LI) was calculated for each histologic category.

CD3 (Dakopatts) and CD8 (Dakopatts) immunostaining was performed by ABC method using Elite Vectastain ABC kit (Vector Laboratories, Inc., CA, USA). After trypsin digestion (0.1% in phosphate-buffered saline (PBS), 37°C, 40 min, Sigma, MO, USA) for CD3 or microwave oven heating (100°C, 15 min) for CD8, the sections were incubated overnight at 4°C with anti-CD3 and CD8 antibodies diluted at 1:100 and 1:200 respectively. Number of CD3- or CD8-positive lymphocytes per ten high power fields was calculated in each case.

### Statistics

Statistical analysis was performed with Statcel software (OMS, Japan). The follow-up period for 35 patients with cancers calculated from the date of initial surgical treatment ranged from 5 to 110 (median 32) months (Table 2). Differences in positive rate among each histologic category and type along with PI values among each histologic category were compared by Fisher's exact

*© 2000 Cancer Research Campaign*

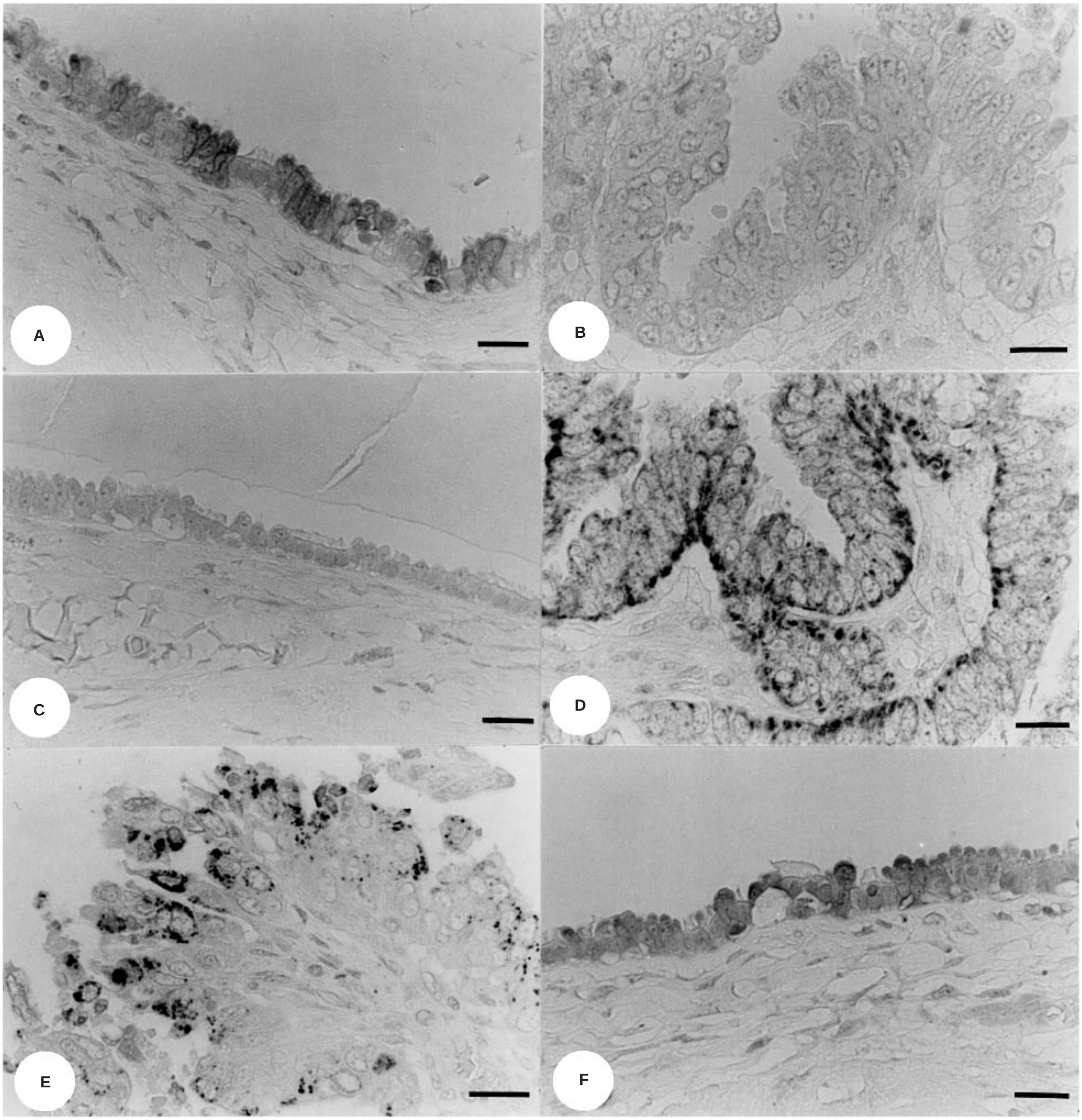

**Figure 1** Immunohistochemical analysis of apoptosis related genes. (**A**) In serous adenoma, epithelial cells were positive for bcl-2 but weak and heterogenous. (**B**) In contrast, tumour cells of serous cystadenocarcinoma were negative for bcl-2. (**C**) Serous adenoma was negative for Fas L. (**D**) In serous cystadenocarcinoma, tumour cells showed granular membranous and cytoplasmic staining of Fas L. (**E**) Serous adenocarcinoma was positive for Fas. Coarse granular intracytoplasmic staining was observed. (**F**) Bax was stained in the cytoplasm of serous adenoma. Bars = 20 μm probability test (Armitage, 1987). Actuarial survival curves of the patients were constructed by the method of Kaplan–Meier (Kaplan and Meier, 1958), and the significance of the differences was estimated by log-rank test. Comparison of age distribution was made by Student's *t*-test, and that of clinical stages by Mann–Whitney *U*-test.

### RESULTS

Results of immunohistochemical studies are summarized in Tables 3 and 4. Although five patients received chemotherapy prior to surgery, this did not considerably affect the stainability of histologic sections.

### Bcl-2 and Bax

In the normal ovary, stromal cells, granulosa cells and surface epithelial cells expressed bcl-2. In the neoplasias, bcl-2 expression was confined in the cytoplasm of epithelial cells, but weak and heterogenous (Figure 1 A, B). Positive rate for bcl-2 was significantly lower in LPM and carcinoma than in adenoma ($P < 0.05$)

© 2000 Cancer Research Campaign

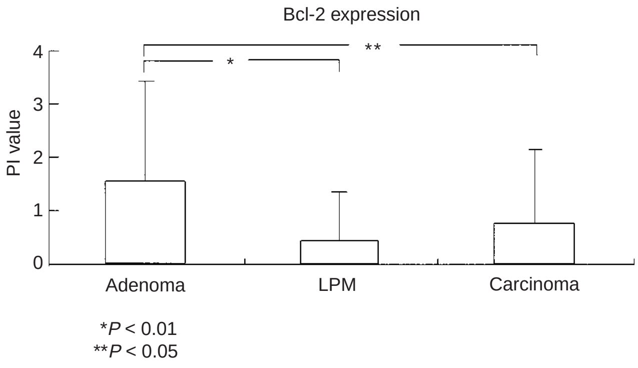

**Figure 2** PI value of bcl-2 in each histologic category. Mean value (± s.d.) were 1.53 ± 1.89 in adenoma, 0.42 ± 0.94 in LPM and 0.74 ± 1.40 in carcinoma. Difference was significant between adenoma and LPM (*P* < 0.01) or adenoma and carcinoma (*P* < 0.05)

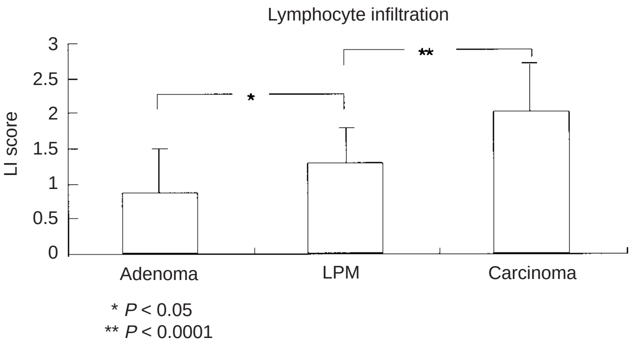

**Figure 4** Score of LI in each category. Mean scores of LI in adenoma, LPM and carcinoma was 0.86 ± 0.64, 1.27 ± 0.52 and 2.00 ± 0.70 respectively. Difference of scores was significant between adenoma and LPM (*P* < 0.05), and LPM and carcinoma (*P* < 0.0001). Bars indicate standard deviation

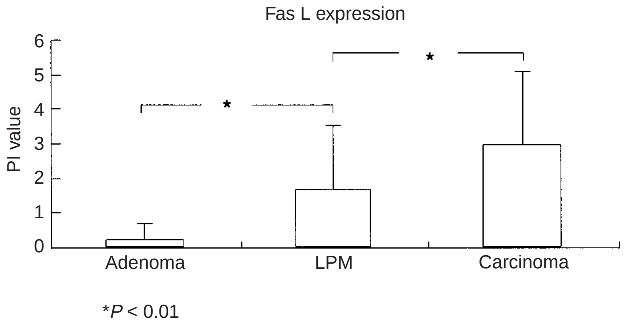

**Figure 3** PI value of Fas L in each histologic category. Mean value (± s.d.) were 0.22 ± 0.48 in adenoma, 1.63 ± 1.88 in LPM and 2.92 ± 1.14 in carcinoma. Difference was significant between adenoma and LPM (*P* < 0.01), LPM and carcinoma (*P* < 0.01), or adenoma and carcinoma (*P* < 0.0001)

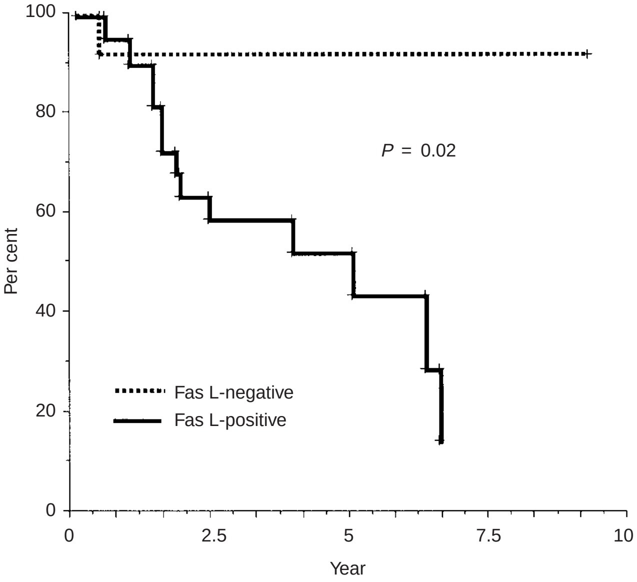

**Figure 5** Patients with Fas L-negative carcinomas showed more favourable prognosis than those with Fas L-positive carcinomas (*P* = 0.02)

(Table 3). PI value of adenoma (1.53 ± 1.89) was significantly higher than that in LPM (0.42 ± 0.94) and carcinoma (0.74 ± 1.40) (Figure 2). Difference in positive rate was significant between serous adenoma and mucinous adenoma ($P < 0.01$), serous LPM and mucinous LPM ($P < 0.05$), and serous carcinoma and clear cell carcinoma ($P < 0.01$) (Table 4).

In the normal tonsil, Bax protein was mainly expressed in germinal centre cells and in basal, parabasal to intermediate cell layer of squamous epithelium (data not shown). Bax protein was weakly expressed in the surface cells of normal ovary (data not shown). There was no significant difference in positive rate for Bax between adenoma, LPM and carcinoma (Table 3 and Figure 1F).

### Fas and Fas ligand

Fas was expressed on the surface of Jurkat cells and surface cells of normal ovary (data not shown). In adenoma, LPM, or carcinoma, not only cell surface but also intra-cytoplasmic staining for Fas was observed. Some tumour cells showed coarse granular intracytoplasmic staining (Figure 1E). There was no prominent difference in the frequency of Fas expression and PI value among each histologic category of ovarian neoplasias. Fas expression was not prominent in infiltrating lymphocytes.

Jurkat cells stimulated with PHA expressed Fas L as clusters near the cell surface. In ovarian neoplasias, Fas L expression was detected not only on the cell surface but also as granular cytoplasmic staining (Figure 1 C, D). Fas L was expressed in only one of 36 cases (3%) with adenoma but 36% and 67% of cases with LPM and carcinoma respectively. The difference in positive rate between each group was significant (Table 3). Difference of PI values between adenoma (0.22 ± 0.48), LPM (1.63 ± 1.88) and carcinoma (2.92 ± 1.14) was also significant (Figure 3). By histological subtype, difference in positive rate was significant between serous LPM and mucinous LPM (Table 4).

### Lymphocyte infiltration and CD3, CD8

Difference in LI values between adenoma (0.86 ± 0.64), LPM (1.27 ± 0.52) and carcinoma (2 ± 0.70) was significant (Figure 4). Twenty cases with serous carcinoma (ten with Fas L expression and ten without) were randomly selected for evaluation of density

 © 2000 Cancer Research Campaign of CD3+ and CD8+ lymphocytes infiltration. In cases with strong Fas L expression, there was a tendency of less infiltration of CD3- and CD8-positive lymphocytes, whereas more infiltration of CD3- and CD8-positive lymphocytes with negative Fas L expression, although the difference was not significant (data not shown).

### Survival analysis

Patients with carcinomas expressing Fas L showed a less favourable 5-year survival rate (44%) than those without Fas L expression (92%) ($P$ = 0.02) (Figure 5). Patients with positive bcl-2 expression showed more favourable prognosis than those without, although the difference was not significant. Any significant correlations between Bax or Fas expressions and survival were not found (data not shown).

### DISCUSSION

In non-small-cell lung carcinoma, breast cancer and ovarian cancer, patients with negative or weak bcl-2 expression showed less favourable prognosis than those with distinct bcl-2 expression (Pezzella et al, 1993; Joensuu et al, 1994; Henriksen et al, 1995). In the present study, carcinoma patients with negative bcl-2 expression also showed poorer prognosis than those with bcl-2 expression, although the difference was not statistically significant. In the study of Henriksen et al (1995), eight of 12 (67%) cases with benign ovarian tumours were positive for bcl-2 expression, while 24 of 50 (48%) with carcinomas were bcl-2-positive. Similar tendency was observed in our study: 14 of 36 (39%) adenoma cases were bcl-2-positive, whereas five of 33 (15%) LPM and 12 of 63 (19%) carcinoma cases were bcl-2-positive. There was a difference in the frequency of bcl-2 expression among each histologic type: none of mucinous tumours were positive for bcl-2, whereas, in serous tumours, 14 of 25 (56%) adenoma, four of 14 (29%) LPM and three of 33 (9%) carcinoma were bcl-2-positive. Although about a half of cases with clear cell carcinoma were bcl-2-positive, bcl-2-positive rate generally decreased along with increase in malignant potential.

Bax is one of the bcl-2 family proteins with 21% homology with bcl-2 protein. Bax heterodimerizes with bcl-2 protein, and the ratio of these two peptides control programmed cell death (Oltvai et al, 1993). There have been few reports suggesting a clinical significance of Bax expression in cancers. Bax expression correlated with favourable prognosis of patients in neuroendocrine lung tumours (Brambilla et al, 1996) or in neuroblastoma (Hoehner et al, 1997). Wehrli et al (1998) reported that there were no significant differences in Bax staining between benign, borderline and malignant ovarian tumours. Our study also did not reveal any correlation between Bax expression and histologic classifications or survival.

Fas is a type I membrane protein that belongs to the TNF receptor family. Human Fas antigen consists of 325 amino acids and transduces death signal conferred by anti-Fas antibody or Fas L. Fas expression was reported in various kinds of human malignancies, including ovarian serous cystadenocarcinoma (Owen-Schaub et al, 1994; Hellquist et al, 1997; Wakahara et al, 1997). In our study, 74% of patients with ovarian neoplasms showed Fas expression without significant difference in the frequency of Fas expression between histologic types.

Fas L is a type II membrane protein consisting of 281 amino acids, that belongs to TNF family protein. Fas L induces cell death via Fas-mediated pathway (Takahashi et al, 1994). It is hypothesized that cancer cells expressing Fas L have an advantage to evade human immune surveillance. In our study, the frequency of cases expressing Fas L increased along with increasing malignant potential. Although statistically not significant, density of CD3+, CD8+ lymphocytes was higher in the serous carcinoma with negative Fas L expression than those with strong Fas L expression. The poorer prognosis of the carcinoma patients expressing Fas L compared to those without was observed in our series. These findings suggest that Fas L-expressing carcinomas induce apoptosis in infiltrating lymphocytes with Fas expression, and escape from immune surveillance. Relatively lower frequency of Fas L expression in the mucinous tumour suggest the presence of different mechanism in growth advantage of this tumour. Follow-up period in our study was relatively short, therefore a further investigation is required to characterize prognostic value of Fas L expression in various ovarian epithelial neoplasms.

A large number of ovarian tumours expressed Fas. Why does apoptosis not occur in Fas-expressing ovarian tumours via co-expressed Fas L? In some reports, Fas L expressed by tumour cells was demonstrated to be functional (Hahne et al, 1996; Shiraki et al, 1997; Ungefroren et al, 1998). So, the dysfunction of Fas-mediated apoptotic system in the tumour cells will explain this phenomenon. This might be supported by the study of Shima et al (1995) showing that two of five myeloma cell lines expressing Fas on their cell surface was insensitive to agonizing anti-Fas monoclonal antibody. There is another possibility that mutated Fas gene contributes to desensitization of Fas system as reported by Landowski et al (1997) or localization of Fas antigen in the cytoplasm not on the cell surface, as reported in oesophageal carcinoma by Hughes et al (1997). In our study, many tumours showed intracytoplasmic staining for Fas antigen along with Fas L. Further investigation will be required to elucidate the question.

### ACKNOWLEDGEMENTS

We thank Drs Masahiko Osawa and Hideo Morino for their kind advice. We also thank Drs Yoshihiko Hoshida, Zhi-ming Dong and Mr Yoshimasa Kabutomori for their technical assistance.

### REFERENCES

Armitage BG (1987) *Statistical Methods in Medical Research*, 2nd edn. Blackwell: Oxford

Barber HRK (1993) *Ovarian Carcinoma. Etiology, Diagnosis, and Treatment*, 3rd edn. Springer-Verlag: New York

Bennett MW, O'Connell J, O'Sullivan GC, Brady C, Roche D, Collins JK and Shanahan F (1998) The Fas counterattack in vivo: apoptotic depletion of tumor-infiltrating lymphocytes associated with Fas ligand expression by human esophageal carcinoma. *J Immunol* **160**: 5669–5675

Brambilla E, Negoescu A, Gazzeri S, Lantuejoul S, Moro D, Brambilla C and Coll JL (1996) Apoptosis-related factors p53, Bcl2, and Bax in neuroendocrine lung tumors. *Am J Pathol* **149**: 1941–1952

Brinton LA and Hoover RN (1992) Epidemiology of gynecologic cancers. In: *Principles and Practice of Gynecologic Oncology*, Hoskins WJ, Perez CA and Young RC (eds), pp. 3–26. JB Lippincott: Philadelphia

Garzetti GG, Ciavattini A, Goteri G, Nictolis MD, Stramazzotti D, Lucarini G and Biagini G (1995) Ki67 antigen immunostaining (MIB 1 monoclonal antibody) in serous ovarian tumors: index of proliferative activity with prognostic significance. *Gynecol Oncol* **56**: 169–174

Hahne M, Rimoldi D, Schröter M, Romero P, Schreier M, French LE, Schneider P, Bornand T, Fontana A, Lienard D, Cerottini JC and Tschopp J (1996) Melanoma cell expression of Fas(Apo-1/CD95) ligand: implications for tumor immune escape. *Science* **274**: 1363–1366

© 2000 Cancer Research Campaign 

Hellquist HB, Olejnicka B, Jadner M, Andersson T and Sederholm C (1997) Fas receptor is expressed in human lung squamous cell carcinomas, whereas bcl-2 and apoptosis are not pronounced: a preliminary report. *Br J Cancer* **76**: 175–179

Henriksen R, Funa K, Wilander E, Bäckström T, Ridderheim M and Öberg K (1993) Expression and prognostic significance of platelet-derived growth factor and its receptors in epithelial ovarian neoplasms. *Cancer Res* **53**: 4550–4554

Henriksen R, Wilander E and Öberg K (1995) Expression and prognostic significance of Bcl-2 in ovarian tumours. *Br J Cancer* **72**: 1324–1329

Hockenbery D, Nuñez G, Milliman C, Shreiber RD and Korsmeyer SJ (1990) Bcl-2 is an inner mitochondrial membrane protein that blocks programmed cell death. *Nature* **348**: 334–336

Hoehner JC, Gestblom C, Olsen L and Påhlman S (1997) Spatial association of apoptosis-related gene expression and cellular death in clinical neuroblastoma. *Br J Cancer* **75**: 1185–1194

Hughes SJ, Nambu Y, Soldes OS, Hamstra D, Rehemtulla A, Iannettoni MD, Orringer MB and Beer DG (1997) Fas/APO-1 (CD95) is not translocated to the cell membrane in esophageal adenocarcinoma. *Cancer Res* **57**: 5571–5578

Joensuu H, Pylkkänen L and Toikkanen S (1994) Bcl-2 protein expression and long-term survival in breast cancer. *Am J Pathol* **145**: 1191–1198

Kaplan EL and Meier P (1958) Non-parametric estimation from incomplete observations. *J Am Stat Ass* **53**: 457–481

Katsube Y, Berg JW and Silverberg SG (1982) Epidemiologic pathology of ovarian tumors: a histopathologic review of primary ovarian neoplasms diagnosed in the Denver Standard Metropolitan Statistical Area, 1 July–31 December 1969 and 1 July–31 December 1979. *Int J Gynecol Pathol* **1**: 3–16

Koonings PP, Campbell K, Mishell DR and Grimes DA (1989) Relative frequency of primary ovarian neoplasms: a 10-year review. *Obstet Gynecol* **74**: 921–926

Landowski TH, Qu N, Buyuksal I, Painter JS and Dalton WS (1997) Mutations in the Fas antigen in patients with multiple myeloma. *Blood* **90**: 4266–4270

McDonnell TJ, Deane N, Platt FM, Nunez G, Jaeger U, Mckearn JP and Korsmeyer SJ (1989) Bcl-2 immunoglobulin transgenic mice demonstrate extended B cell survival and follicular lymphoproliferation. *Cell* **57**: 79–88

Müllauer L, Mosberger I and Chott A (1998) Fas ligand expression in nodal non-Hodgkin's lymphoma. *Mod Pathol* **11**: 369–375

Nagata S and Golstein P (1995) The Fas death factor. *Science* **267**: 1449–1456

Niehans GA, Brunner T, Frizelle SP, Liston JC, Salerno CT, Knapp DJ, Green DR and Kratzke RA (1997) Human lung carcinomas express Fas ligand. *Cancer Res* **57**: 1007–1012

Oltvai ZN, Milliman CL and Korsmeyer SJ (1993) Bcl-2 heterodimerizes in vivo with a conserved homolog, Bax, that accelerates programmed cell death. *Cell* **74**: 609–619

Omura GA, Brady MF, Homesley HD, Yordan E, Major FJ, Buchsbaum HJ and Park RC (1991) Long-term follow-up and prognostic factor analysis in advanced ovarian carcinoma: the Gynecologic Oncology Group experience. *J Clin Oncol* **9**: 1138–1150

Owen-Schaub LB, Radinsky R, Kruzel E, Berry K and Yonehara S (1994) Anti-Fas on nonhematopoietic tumors: levels of Fas/APO-1 and bcl-2 are not predictive of biological responsiveness. *Cancer Res* **54**: 1580–1586

Pezella F, Turley H, Kuzu I, Tungekar MF, Dunnill MS, Pierce CB, Harris A, Gatter KC and Mason DY (1993) Bcl-2 protein in non-small cell lung carcinoma. *N Engl J Med* **329**: 690–694

Rodenburg CJ, Cornelisse CJ, Heintz PAM, Hermans JO and Fleuren GJ (1987) Tumor ploidy as a major prognostic factor in advanced ovarian cancer. *Cancer* **59**: 317–323

Shima Y, Nishimoto N, Ogata A, Fujii Y, Yoshizaki K and Kishimoto T (1995) Myeloma cells express Fas antigen/APO-1 (CD95) but only some are sensitive to anti-Fas antibody resulting in apoptosis. *Blood* **85**: 757–764

Shiraki K, Tsuji N, Shioda T, Isselbacher KJ and Takahashi H (1997) Expression of Fas ligand in liver metastases of human colonic adenocarcinomas. *Proc Natl Acad Sci USA* **94**: 6420–6425

Slamon DJ, Godolphin W, Jones LA, Holt JA, Wong SG, Keith DE, Levin WJ, Stuart SG, Udove J, Ullrich A and Press MF (1989) Studies of the HER-2/neu proto-oncogene in human breast and ovarian cancer. *Science* **244**: 707–712

Strand S, Hofmann WJ, Hug H, Müller M, Otto G, Strand D, Mariani SM, Stremmel W, Krammer PH and Galle PR (1996) Lymphocyte apoptosis induced by CD95 (APO-1/Fas) ligand-expressing tumor cells – a mechanism of immune evasion? *Nature Med* **2**: 1361–1366

Takahashi T, Tanaka M, Inazawa J, Abe T, Suda T and Nagata S (1994) Human Fas ligand: gene structure, chromosomal location and species specificity. *Int Immunol* **6**: 1567–1574

Tanaka M, Suda T, Haze K, Nakamura N, Sato K, Kimura F, Motoyoshi K, Mizuki M, Tagawa S, Ohga S, Hatake K, Drummond AH and Nagata S (1996) Fas ligand in human serum. *Nat Med* **2**: 317–322

Ungefroren H, Voss M, Jansen M, Roeder C, Henne-Bruns D, Kremer B and Kalthoff H (1998) Human pancreatic adenocarcinomas express Fas and Fas ligand yet are resistant to Fas-mediated apoptosis. *Cancer Res* **58**: 1741–1749

Wakahara Y, Nawa A, Okamoto T, Hayakawa A, Kikkawa F, Suganuma N, Wakahara F and Tomoda Y (1997) Combination effect of anti-Fas antibody and chemotherapeutic drugs in ovarian cancer cells in vitro. *Oncology* **54**: 48–54

Wehrli BM, Krajewski S, Gascoyne RD, Reed JC and Gilks CB (1998) Immunohistochemical analysis of bcl-2, bax, mcl-1, and bcl-X expression in ovarian surface epithelial tumors. *Int J Gynecol Pathol* **17**: 255–260

*© 2000 Cancer Research Campaign*
